# Axillary Versus Subclavian Venous Access for Permanent Pacemaker Implantation: Complications, Evolving Techniques and Practical Recommendations

**DOI:** 10.3390/medicina61122173

**Published:** 2025-12-06

**Authors:** Georgios Aletras, Spyridon Stavratis, Ermis Hoda, Emmanuel Rogdakis, Emmanuel Koutalas, Emmanuel Kanoupakis, Emmanuel Foukarakis

**Affiliations:** 1 Department of Cardiology, Venizelio General Hospital of Heraklion, 71409 Heraklion, Greece; spirostavratis@gmail.com (S.S.); ermishoda@gmail.com (E.H.); rogdakisem@gmail.com (E.R.); mfouk@hotmail.com (E.F.); 2 School of Medicine, University of Crete, 70013 Heraklion, Greece; 3 Department of Cardiology, University General Hospital of Heraklion, 71500 Heraklion, Greece; emmkout@gmail.com (E.K.); kanoup@gmail.com (E.K.)

**Keywords:** pacemaker, cardiac implantable electronic devices, venous access, axillary vein, subclavian vein, complications, ultra-sound guided

## Abstract

Venous access selection represents a pivotal aspect of permanent pacemaker implantation, directly influencing both procedural safety and long-term device performance. Although the subclavian vein has been traditionally favored, its use is associated with notable risks, including pneumothorax, hemothorax, and crush syndrome. In contrast, axillary access has gained prominence, supported by observational studies and a small number of randomized trials demonstrating reduced pneumothorax rates, fewer arterial punctures, and potentially fewer long-term mechanical lead failures, particularly when ultrasound guidance is used. However, these findings are derived from heterogeneous studies with variable definitions, imaging protocols and operator experience, highlighting that evidence quality remains moderate. Contemporary advances—such as routine ultrasound guidance, micropuncture techniques, and structured training—have improved procedural consistency and safety, a trend reflected in recent guidelines. This review provides a focused appraisal of complication patterns, anatomical differences and patient-specific considerations to support individualized and evidence-aligned venous access selection in modern clinical practice.

## 1. Introduction

Pacemaker implantation is a widely performed and generally safe procedure for managing bradyarrhythmias. The global number of permanent pacemaker implantations has steadily increased, with current estimates approaching one million devices annually. High-degree atrioventricular block (AVB) and sinus node dysfunction remain the most common indications for pacing therapy [1,2].

A key first step in pacemaker implantation is obtaining safe venous access for lead placement in the right ventricle or atrium. As patients live longer and more often need future procedures like device upgrades or lead revisions, the choice of access route carries important long-term implications [3,4]. Traditionally, the subclavian vein has been the most widely used access site due to its predictable anatomical location and operator familiarity with the Seldinger technique. However, subclavian access is associated with several well-recognized complications, including pneumothorax, hemothorax, inadvertent arterial puncture, and lead failure (crush syndrome). These limitations have prompted growing interest in alternative techniques [4,5,6,7,8].

The axillary vein, which courses more laterally and extrathoracically, has become an increasingly utilized alternative. Several comparative studies—primarily observational studies—suggest that axillary access may be associated with a lower risk of intrathoracic complications and mechanical lead failure [6,7,9,10]. However, randomized prospective data remain limited, and concerns persist regarding technical complexity and operator experience [11]. Complication risk increases with device complexity and is higher in upgrades or lead revisions compared to de novo implantations. Rates are also closely linked to operator and center experience, with significantly more complications seen in low-volume centers or emergency procedures [2,12,13].

In recent years, the focus has shifted toward safer and image-guided strategies that balance procedural efficiency with complication prevention. These developments have paralleled broader efforts to refine access techniques and improve procedural consistency through modern imaging tools and structured operator training. This review provides a critically appraised comparison of subclavian and axillary approaches and integrates evolving imaging techniques, operator training considerations, and patient-specific factors to guide contemporary clinical decision-making.

## 2. Literature Review Strategy

To inform this narrative review, we conducted a targeted literature search using PubMed/MEDLINE, Scopus, and Google Scholar from database inception to 15 July 2025. Search terms included combinations of “pacemaker,” “cardiac implantable electronic device (CIED)”, “venous access,” “subclavian,” “axillary,” “cephalic,” “ultrasound-guided,” “lead complications,” and “lead failure”. We restricted our search to human studies in adults, published in English and focusing on transvenous pacemaker or CIED implantation.

We prioritized:

▪ Randomized and non-randomized comparative studies of axillary versus subclavian access;
▪ Large observational cohorts and national registries;
▪ Meta-analyses and systematic reviews;
▪ Guideline and consensus documents from professional societies such as the European Society of Cardiology (ESC) and American College of Cardiology/American Heart Association/Heart Rhythm Society (ACC/AHA/HRS).

We excluded pediatric-only studies, purely surgical epicardial systems and studies without a clearly defined venous access technique. Case reports were included only when illustrating rare complications or providing mechanistic insight, but they were not used for comparative evaluation. Additional references were identified through the reference lists of key articles. Given the narrative scope of this review, no formal PRISMA flow diagram or meta-analytic pooling was performed; however, we aimed to provide transparent methodological reporting and highlight the quality, strengths and limitations of included studies.

## 3. Anatomical and Technical Considerations

Selecting an appropriate venous access route requires a clear understanding of the anatomical and technical nuances that differentiate subclavian and axillary entry sites. Although these veins form a continuous pathway, their relationship to surrounding structures, suitability for imaging guidance and the mechanical implications for long-term lead performance differ meaningfully. These distinctions directly affect procedural safety, complication patterns and long-term integrity. The following sections provide a practical, comparative overview to support informed decision-making in daily clinical practice.

### 3.1. Subclavian Vein Access

The subclavian vein has served as the standard venous access for transvenous pacemaker implantation for decades [6,14]. Its consistent anatomical course beneath the clavicle and direct trajectory toward the superior vena cava allow rapid access using the Seldinger technique under fluoroscopic guidance. In difficult cases or during multiple lead insertions contrast venography may provide further anatomical delineation. These features contribute to its long-standing popularity and high procedural success rates [15].

Despite these advantages, subclavian access is associated with several well-recognized risks due to its close proximity to the pleura, lung apex and subclavian artery. Complications include pneumothorax, hemothorax, inadvertent arterial cannulation and brachial plexus injury. A key long-term concern is lead crush syndrome, caused by compression of the lead between the clavicle and first rib, leading to insulation damage or conductor fracture—more frequently affecting younger and more active patients [2,5,11,16,17]. Although endothelial trauma during puncture has been implicated in venous stenosis, current evidence has not clearly demonstrated a major difference in long-term venous obstruction between subclavian and axillary routes [13,18,19].

### 3.2. Axillary Vein Access

**Figure 1:**
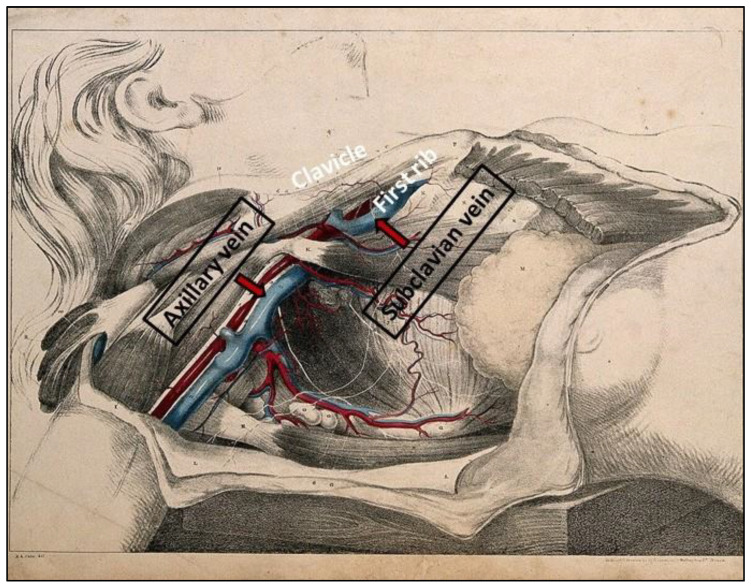
Subclavian and axillary venous anatomy relevant to pacemaker implantation. Illustration showing the axillary vein as an extrathoracic continuation of the subclavian vein and its relationship with the clavicle and first rib. Adapted from a colored lithograph by G.E. Madeley after A.A. Cane, 1834, Wellcome Collection [24]. Licensed under CC BY 4.0 (https://creativecommons.org/licenses/by/4.0/). URL accessed on 3 November 2025. Modifications (labels added) by the authors.

The axillary vein, a lateral continuation of the subclavian vein, provides an extrathoracic access route that avoids clavicle-first rib space (Figure 1). Its more lateral position is associated with a lower risk of intrathoracic complications [20,21]. The axillary vein can be accessed using various methods, including fluoroscopic guidance (e.g., “first rib technique”), ultrasound guidance, which allows direct visualization of the vein and surrounding structures and anatomical landmarks. Access using landmarks alone is less reliable than imaging-guided [20,22].

In recent years, ultrasound-guided axillary access has become increasingly adopted due to its ability to improve first-pass success, reduce arterial puncture, and pneumothorax, and markedly reduce reliance on fluoroscopy. The more parallel lead entry angle through the axillary vein may reduce mechanical stress on pacing leads and potentially enhance long-term lead performance (Table 1) [21,23].

## 4. Procedural Success and Learning Curve

Both subclavian and axillary access achieve high success rates when performed by experienced operators; however, the performance profile of each technique differs, particularly in the modern era of routine ultrasound guidance [22,25].

### 4.1. Fluoroscopy-Guided Access

Fluoroscopy-guided puncture remains the most widely used technique, offering high procedural success rates, especially for experienced operators. Nevertheless, this method carries drawbacks, including radiation exposure and occasional need for contrast venography, which may be undesirable in patients with renal dysfunction [23].

### 4.2. Ultrasound-Guided Access

**Figure 2:**
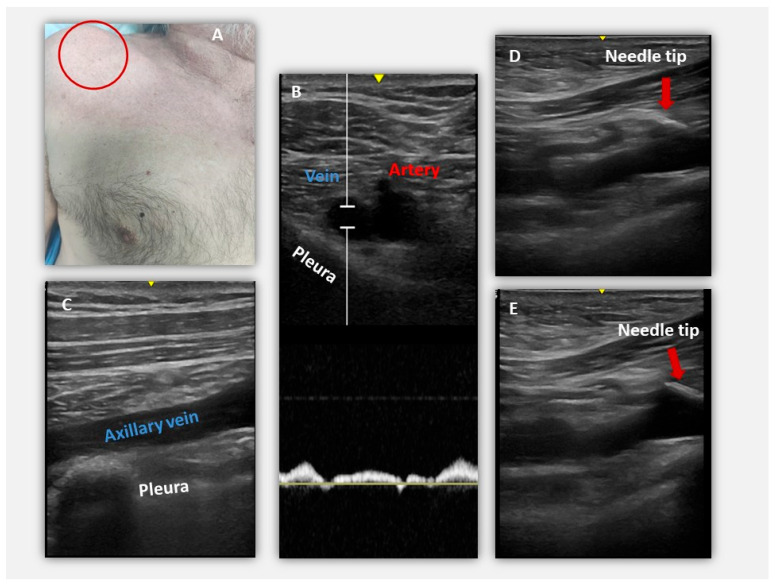
Ultrasound-guided axillary venous access: key landmarks and needle trajectory. (**A**) Surface anatomy and probe position. (**B**) Transverse view showing axillary vein (blue), axillary artery (red), and pleura. (**C**) Clear visualization of the axillary vein with the pleura deep to the vessel. (**D**,**E**) Real-time visualization of the needle tip advancing toward the axillary vein. These images demonstrate safe needle alignment, avoidance of pleural structures, and advantages of direct ultrasound guidance. Panels obtained from anonymized ultrasound images acquired during routine pacemaker implantation; no patient-identifying information is present.

Ultrasound-guided access enables real-time visualization of target vessel, surrounding anatomy, and needle trajectory (Figure 2). Its benefits include lower incidence of arterial puncture and pneumothorax, reduced reliance on anatomical landmarks and improved first-pass success rates, as reported in multiple studies. These advantages are most pronounced in axillary access, where the vessel is more superficial and not obscured by the clavicle [21,23,25,26,27].

### 4.3. Operator Learning Curve

Operator familiarity is a critical factor influencing the choice of technique. The subclavian approach is more commonly taught and performed, leading to broader comfort among general cardiologists and trainees. In contrast, the axillary approach, especially when performed under ultrasound guidance, requires more time and training to achieve procedural proficiency. Anatomical variability, vessel depth, and the need for hand–eye coordination during ultrasound-guided puncture all contribute to initial challenges [28]. However, once procedural proficiency is achieved, ultrasound-guided axillary access has been reported to offer comparable—or in some cases superior—safety to subclavian access, particularly for arterial puncture and pneumothorax [25,29].

### 4.4. Technological and Training Advances

Modern refinements—such as the use of 21-gauge micropuncture needles, high-frequency linear probes and intra-pocket ultrasound—have improved the safety profile of axillary access, offering strict control during vessel entry and minimizing procedural time and the risk of vascular or pleural injury [27,30,31].

These considerations have important implications for training programs. As prevention of access-related complications becomes a central priority in device implantation, there is increasing recognition of the need to incorporate ultrasound-guided vascular access into cardiology and electrophysiology curricula. Structured teaching and simulation-based practice can support operators in overcoming the initial learning curve and adopting these techniques safely, potentially facilitating wider use of the axillary vein approach where appropriate [23,28].

## 5. Complications

Understanding the complication profile associated with each venous access route is essential for procedural planning and long-term device management. Table 2 provides a structured comparison of complication rates based on contemporary observational studies, randomized trials, and meta-analyses [6,11].

### 5.1. Pneumothorax and Hemothorax

Pneumothorax remains one of the most significant acute complications of transvenous pacemaker implantation, particularly with the subclavian approach. Contemporary series report pneumothorax in 1–3% of subclavian punctures and <1% with axillary access, with the lowest rates when ultrasound guidance is used. Hemothorax and inadvertent arterial puncture are less common but occur when the subclavian/axillary artery is unintentionally accessed [9,11,29,32].

Diagnosis typically relies on chest X-ray, though ultrasound or computed tomography (CT) may be used in clinically unstable patients. Management depends on severity: small, stable cases of pneumothorax may only require observation and oxygen, while larger or symptomatic ones may necessitate chest tube insertion. Hemothorax requires prompt drainage and rarely surgical intervention [15].

### 5.2. Lead Failure and Crush Syndrome

Mechanical lead failure is more frequently observed with subclavian access due to potential compression between the clavicle and the first rib (“crush syndrome”). This may lead to insultation damage or conductor fracture over time [33,34]. Axillary access provides a straighter and less angulated lead path, with observational studies reporting lower rates of chronic lead malfunction [11,32,35].

Diagnosis relies on device interrogation showing abnormal impedance, threshold, or sensing parameters. Radiographic evaluation may assist in identifying structural lead damage. Management typically requires lead revision or replacement [15,33].

### 5.3. Venous Stenosis and Occlusion

Venous stenosis or occlusion occurs in a substantial proportion of patients with transvenous cardiac implantable electronic devices (CIED) systems, especially during upgrades or in the presence of multiple implanted leads. Available studies do not demonstrate a consistent difference between subclavian and axillary access, likely due to heterogeneity in definitions, imaging protocols, and follow-up duration [15,36]. The burden of implanted leads, prior instrumentation, and endothelial trauma are stronger predictors of stenosis than the puncture site itself [36,37].

Most cases remain asymptomatic due to collateral formation, but in cases of severe obstruction—particularly involving the superior vena cava (SVC)—patients may develop edema, dyspnea or venous congestion [38,39]. Management depends on the severity of obstruction and the clinical context. In asymptomatic cases, no intervention may be required. In symptomatic patients or those undergoing reintervention, strategies may include venoplasty, lead extraction, or recanalization with stenting, although success can be variable [38,40,41,42].

### 5.4. Arterial Injury and Hematoma

Both subclavian and axillary venous access techniques carry a risk of arterial puncture and subsequent hematoma formation. Subclavian access traditionally exhibits higher rates when performed without imaging, largely because misplacement may go unrecognized beneath the clavicle [15].

In contrast, the axillary vein lies immediately adjacent to the axillary artery, and some observational studies reported higher arterial puncture rates with axillary access when performed using fluoroscopy alone. However, ultrasound guidance—especially when combined with a 21-gauge micropuncture needle—can reduce this risk by allowing real-time visualization of the vein, artery, needle tip, and pleural line, while minimizing the consequences of inadvertent arterial entry. Randomized and prospective studies consistently demonstrate significantly lower rates of clinically relevant arterial injury with ultrasound-guided axillary access, even in anticoagulated patients [25,27,31,43].

Management typically involves immediate manual compression; most hematomas are self-limiting. Imaging is reserved for expanding hematomas or suspected neurovascular compromise [44,45].

### 5.5. Infection

The risk of infection following CIED implantation is influenced more by procedural and patient-related factors than by the choice of venous access route. No consistent differences in infection rates have been shown between subclavian and axillary approaches. Instead, comorbidities such as diabetes, renal dysfunction, heart failure, and COPD, along with procedural factors like temporary pacing, early reintervention, and prolonged operative time, play a more significant role [46,47].

Diagnosis relies on clinical evaluation, blood cultures, inflammatory markers, and imaging when needed, though normal inflammation markers do not exclude infection [2,46]. Preventive strategies remain essential. A single dose of preprocedural cefazolin has been shown to reduce the risk of infection significantly, and current guidelines recommend administering antibiotics within one hour of incision, along with sterile technique and chlorhexidine–alcohol for skin preparation [2,15]. When ultrasound-guided venous access is used, the ultrasound probe must be disinfected appropriately and covered with a sterile sheath to avoid contamination of the operative field [48,49]. Management depends on the extent of infection. Localized cases, e.g., superficial incisional infections, may respond to antibiotics, but the majority of confirmed device infections require complete system extraction and targeted intravenous therapy [15,46,50].

### 5.6. Radiation Exposure

Radiation exposure is an important procedural consideration, particularly in centers seeking to minimize cumulative operator and patient dose. Subclavian access performed under fluoroscopic guidance typically involves modest radiation exposure. Axillary access may require longer fluoroscopy during the learning curve; however, ultrasound-guided axillary puncture substantially reduces or eliminates fluoroscopy use. Therefore, the total radiation dose is more closely related to imaging strategy than to the venous access route itself [25,29,31,43].

**Table 2:** Comparative overview of complication rates for subclavian versus axillary access. Approximate ranges based on pooled data from contemporary observational cohorts, registries, and selected randomized studies. Rates vary across populations, definitions, and operator experience; therefore, values should be interpreted as indicative rather than definitive.

Parameter	Subclavian Access	Axillary Access	Data Source
Pneumothorax	1–3%	<1%	Kirkfeldt et al. [5]; Sharma et al. [10]; Liu et al. [9]; Vitali et al. [25]
Lead Fracture/Crush Syndrome	2–6%	<2%	Chan et al. [6], Kim et al. [35]; Liu et al. [9]
Arterial Puncture/Hematoma	1–4%	1–17%	Vitali et al. [25]; Maffè et al. [7]; Liccardo et al. [21]
		Significantly reduced with ultrasound	
Hemothorax	<1–1.5%	Rare (<0.5%)	Vitali et al. [25]; Liu et al. [9]
Infection	Similar	Similar	Blomström-Lundqvist C et al. [46]; Kirkfeldt et al. [47]
	Not influenced by access site		
First-pass success rate	40–96%	60–97%	Leventopoulos et al. [27]; Chan et al. [6]; Sharma et al. [10]; Liu et al. [9]; Liccardo et al. [21]
Radiation time	Higher when venography used	Markedly reduced with ultrasound	Vitali et al. [25]; Migliore et al. [22]

To further contextualize these findings and provide an evidence-based comparison across studies, Table 3 synthesizes major prospective, randomized, and large observational cohorts evaluating venous access strategies. By outlining study design, sample size, primary outcomes, and methodological limitations, this table highlights the substantial heterogeneity in the available literature. Such variability underscores the need for cautious interpretation of pooled results and reinforces the importance of high-quality multicenter trials to guide definitive clinical recommendations.

## 6. Special Populations

Selecting the optimal venous access site for pacemaker implantation may be influenced by specific patient characteristics and comorbidities. Certain anatomical or clinical factors may favor an extrathoracic approach, imaging-guided techniques, or non-transvenous alternatives.

### 6.1. Patients with Chronic Kidney Disease (CKD)

In patients with CKD —particularly those approaching end-stage renal disease (ESRD) or already on dialysis—preserving central venous patency is essential because transvenous leads can contribute to venous stenosis that later compromises arteriovenous (AV) fistula creation. While no randomized data directly compare axillary and subclavian access in this specific population, the general principle remains conservation of venous pathways, particularly in patients with existing or planned AV fistulas [51,52].

In selected patients, non-transvenous alternatives—including leadless pacemakers, epicardial systems or subcutaneous ICDs—offer meaningful advantages by bypassing the venous system entirely. These options may also reduce infection risk, which is elevated in CKD due to impaired immunity and frequent device handling. Ultimately, venous access planning in CKD should be individualized, ideally coordinated with nephrology, to balance pacing needs with long-term vascular access preservation [52,53,54,55].

### 6.2. Elderly/Frail and High Bleeding-Risk Patients

Older patients frequently present with multiple comorbidities and a higher pre-procedural risk profile, which increases the likelihood of complications during pacemaker implantation [56,57]. Notably, in a large Danish registry study by Kirkfeldt et al., advancing age was independently associated with a higher risk of pneumothorax following dual-chamber device implantation [5].

This vulnerability is even more pronounced in patients with coagulopathy or elevated bleeding risk, where inadvertent arterial injury during an intrathoracic puncture can lead to expanding hematoma or hemothorax. In such scenarios, the axillary vein, particularly when accessed under real-time ultrasound guidance improves control of needle advancement and it has been associated with very low rates of arterial puncture and clinically significant hematoma, even among patients receiving anticoagulation [7,25,29,58]. 

For elderly, frail or high-bleeding risk individuals, the combination of a compressible access site, a lower risk of intrathoracic complications and precise needle guidance makes the axillary approach an attractive option. Successful implementation nevertheless depends on operator experience, familiarity with ultrasound techniques, and institutional support. Tailoring the venous access strategy to patient-specific factors—frailty status, bleeding risk, chest anatomy, and procedural complexity—remains essential to minimizing perioperative morbidity in this high-risk group [27,31,43,59].

### 6.3. Patients with Prior Device Implantation or Lead Revisions

Patients undergoing device upgrades, lead revisions, or generator replacements frequently exhibit venous stenosis or occlusion, complicating vascular access. Preprocedural imaging is therefore essential [60].

Ultrasound-guided axillary vein access may offer additional flexibility by helping identify patent lateral venous segments, even when more central segments are narrowed. When venous obstruction is more advanced, non-transvenous alternatives—including leadless pacemakers and subcutaneous implantable cardioverter defibrillators (S-ICDs)—serve as effective first-line solutions for preventing further venous compromise [55,61,62]. Transvenous lead extraction may also be considered, especially in younger patients requiring multiple new leads, but should be performed only in high-volume centers with surgical backup due to procedural complexity and inherent risks. Nevertheless, contemporary series—including recent data demonstrating safe same-day discharge after uncomplicated extractions—indicate that the overall management of extraction-related complications has improved markedly in recent years [63,64].

While the current comparison between axillary and subclavian access focuses on conventional transvenous techniques, these newer technologies represent an important paradigm shift in managing complex reintervention cases and should be considered first-line when clinically appropriate [55,61].

### 6.4. Obese Patients or Those with Challenging Anatomy

Patients with obesity or altered anatomical landmarks present difficulties during venous access. Ultrasound-guided axillary access improves visualization of vascular structures and helps avoid inadvertent arterial or pleural injury, though excessive soft tissue may reduce image quality in individuals with high body mass index (BMI) [31,59,65]. Techniques such as intra-pocket ultrasound-guided axillary access, as demonstrated in the ACCESS trial, help overcome these limitations by allowing visualization of the axillary vein from within the device pocket. This method has been shown to improve success rates while maintaining a favorable safety profile [30].

### 6.5. Leadless Pacing and Implication for Venous Access

Leadless pacemakers (LPMs) have become an important alternative in patients for whom transvenous access carries elevated procedural or long-term risk. They are particularly useful in individuals needing single-chamber ventricular pacing who have high infection or bleeding risk, limited upper-extremity venous access, prior tricuspid valve surgery, or very low BMI where a pocket may be problematic. Observational data consistently show lower rates of pocket- and lead-related complications—including infection, hematoma, pneumothorax, and lead failure—compared with transvenous systems. Although not suited for patients requiring biventricular pacing, LPMs may be advantageous in those with intermittent pacing needs or when avoiding a transvenous lead is clinically desirable. Dual-chamber leadless systems remain selectively used due to higher cost and evolving evidence [55,61,66]. The femoral approach is standard, but an internal jugular route has been successfully used in cases where femoral delivery is not possible (e.g., inferior vena cava filters or unfavorable anatomy), expanding applicability in complex access scenarios [66].

Taken together, these subgroups highlight the importance of tailoring venous access selection to individual risk profiles. In patients with CKD, preserving central venous patency and considering non-transvenous options may be advantageous. In elderly, frail, or high-bleeding-risk individuals, ultrasound-guided axillary access offers a compressible extrathoracic entry site that may mitigate the consequences of vascular injury. In patients with prior devices or venous stenosis, ultrasound guidance can help identify suitable windows for axillary puncture, while leadless or subcutaneous systems should be considered when conventional access is limited. Finally, in patients with obesity or those with challenging anatomy, ultrasound-based techniques—including intra-pocket approaches—improve visualization and procedural success. Integrating these patient-specific considerations into pre-procedural planning supports more consistent, individualized, and safer access selection in contemporary practice [26,29,31,43].

## 7. Current Guidelines and Practice Patterns

The selection of venous access during CIED implantation is a critical procedural decision that directly influences both short- and long-term outcomes. The 2021 European Society of Cardiology (ESC) Guidelines on cardiac pacing and resynchronization therapy recommend the cephalic and axillary vein as the preferred routes over the traditional subclavian vein, primarily to reduce the risk of complications. This recommendation is graded as Class IIa, Level of Evidence C, reflecting expert consensus in the absence of large-scale randomized trials. Notably, the ESC guidelines highlight a 7.8-fold increased risk of pneumothorax with subclavian vein puncture compared to extrathoracic routes. Axillary access—particularly under ultrasound guidance—is increasingly considered for its favorable safety profile, reduced fluoroscopy requirements and high first pass-success rates in contemporary practice [2,5,21,25].

The cephalic vein cutdown remains the technique with the lowest access-related complication rate, but its feasibility can be limited by anatomical variability, smaller vessel caliber, and lower rates of successful lead advancement. These challenges may prolong procedural time and reduce its suitability in urgent or complex cases, emphasizing the need for operator training in surgical venous access techniques [3,6].

In contrast, the most recent American guidelines (ACC/AHA/HRS) do not specify a preferred venous access route. While they offer detailed recommendations for procedural safety and infection prevention, no formal preference is expressed among subclavian, axillary, or cephalic access—likely reflecting heterogeneous practice patterns and operator training across U.S. centers [67,68].

The ESC also stresses the role of pre-procedural imaging (e.g., venography or chest CT) in patients with prior leads or suspected venous obstruction. When superior venous access is not feasible, alternative options—such as transfemoral temporary pacing, surgical epicardial leads, or non-transvenous systems including leadless pacemakers and subcutaneous ICDs—are recommended, particularly in patients with central venous obstruction or elevated infection risk [2].

Overall, current ESC recommendations reflect a clear shift toward extrathoracic and imaging-guided techniques, particularly for high-risk subgroups. However, implementation remains dependent on institutional protocols and operator expertise, underscoring the need for guideline-aligned training, investment in ultrasound infrastructure, and broader adoption of modern access techniques [2,8].

**Figure 3:**
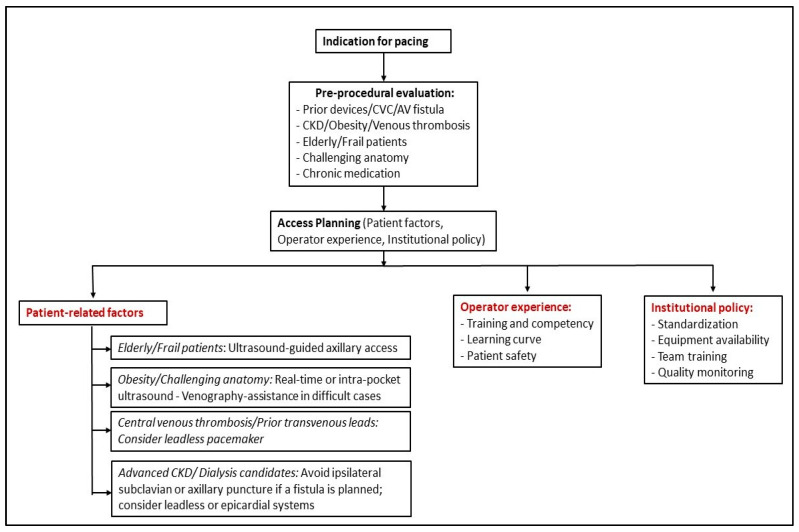
Suggested clinical decision pathway for venous access selection during permanent pacemaker implantation. The algorithm integrates patient-related factors (e.g., chronic kidney disease, obesity, challenging anatomy, frailty, or prior devices), operator experience, and institutional policy to guide individualized access planning. Ultrasound-guided axillary puncture is preferred in most cases, while alternative strategies—including leadless pacemakers or epicardial systems—may be indicated in patients with central venous obstruction or advanced renal disease. Abbreviations: AV: Arteriovenous; CKD: Chronic kidney disease; CVC: Central venous catheter.

A structured, patient-centered approach is essential when selecting the optimal venous access route for pacemaker implantation. Figure 3 summarizes a proposed clinical decision pathway incorporating patient characteristics, operator experience, and institutional policies.

## 8. Limitations in the Literature and Future Perspectives

Despite the widespread use of both subclavian and axillary approaches for transvenous pacemaker implantation, the available literature is predominantly composed of observational studies and registry data, with a notable lack of randomized controlled trials (RCTs) directly comparing these access routes. This limits the strength of recommendations in current guidelines, as reflected by the Class IIa, Level of Evidence C rating assigned by the 2021 ESC Guidelines. Likewise, the. ACC/AHA/HRS guidelines do not endorse a specific venous access route, further highlighting the absence of consensus-driven, high-quality comparative evidence in this area [2,67].

A critical limitation across many studies is the presence of significant heterogeneity and the failure to account for key confounders such as operator experience, body habitus, and institutional volume—all of which can meaningfully influence complication rates such as pneumothorax, hematoma, or lead dislodgement. These factors complicate direct comparisons and likely explain the variability seen across observational cohorts [2,12]. 

Although recent studies support the safety and feasibility of ultrasound-guided axillary access, prospective multicenter validation remains scarce. Adoption in routine practice is still variable, often limited by insufficient training, lack of structured ultrasound curricula, and differences in institutional infrastructure. The learning curve associated with ultrasound-guided techniques is frequently underrecognized in the literature, despite evidence that operator proficiency markedly reduces complication rates [8,12,28].

Data from anatomically complex or clinical challenging subgroups—such as patients with morbid obesity, venous occlusion or prior device revisions—are limited. The increasing use of leadless pacemakers and subcutaneous ICDs represents an important shift in device therapy, especially in patients with central venous occlusion or high infection risk. However, these newer technologies remain largely absent from randomized controlled trials directly comparing pacing strategies and their role as first-line solutions in specific populations continues to evolve [66].

Finally, registry data—although valuable for capturing real-world outcomes—are inherently limited by reporting bias, incomplete data capture, and lack of procedural granularity. Future research should prioritize large-scale prospective studies with standardized outcome definitions, long-term follow-up, and inclusion of both conventional and leadless device technologies. Incorporating artificial intelligence (AI) for procedural planning, risk stratification, and operator training also represents a promising but underexplored avenue [12,28,69]. Looking ahead, cross-disciplinary advances in electrophysiology, critical care, and structural interventional cardiology are expected to further shape access strategies. The growing use of ultrasound-guided extrathoracic techniques, together with the expanding role of axillary arterial access in structural interventions such as transcatheter aortic valve replacement (TAVR), large-bore mechanical circulatory support, and extracorporeal membrane oxygenation (ECMO), highlights a broader shift toward axillary-based vascular approaches in selected patients. Continued innovation, standardized training, and multicenter prospective evaluation will be essential to define the optional role of these evolving access techniques in contemporary practice [70,71,72,73,74].

## 9. Conclusions

The choice of venous access during permanent pacemaker implantation directly influences procedural safety and long-term performance. Among available techniques, the axillary vein approach—particularly when performed under ultrasound guidance and using micropuncture equipment—has demonstrated a favorable safety profile in observational studies with lower reported rates of pneumothorax, arterial injury and mechanical lead complications compared to the traditional subclavian access. These advantages, however, are derived largely from non-randomized data and should be interpreted within the context of heterogeneous study populations

The incorporation of imaging guidance has contributed to reducing access-related complications and minimizing radiation exposure, with benefits becoming more pronounced as operator proficiency increases. At the same time, emerging technologies such as leadless pacemakers and subcutaneous ICDs have expanded the therapeutic landscape for patients at high risk of infection, venous obstruction, or complex reinterventions.

As evidence continues to evolve, venous access strategies should be individualized according to patient anatomy, comorbidities, procedural complexity, and institutional expertise. A progressive shift toward extrathoracic, ultrasound-guided approaches—supported by growing experience across electrophysiology, critical care, and structural interventional specialties—is expected to further enhance safety and procedural consistency in contemporary practice.

## Figures and Tables

**Table 1:** Comparison of Subclavian and Axillary Venous Access for Permanent Pacemaker Implantation. Key anatomical, procedural, and complication-related features of the two most commonly used venous access routes for transvenous pacemaker lead placement [6,21,23].

Feature	Subclavian Access	Axillary Access
Anatomical site	Infraclavicular, Intrathoracic	Lateral, Extrathoracic
Guidance method	Landmarks, Fluoroscopy	Fluoroscopy, Ultrasound, Landmarks
Risk of pneumothorax	Higher	Lower
Lead crush syndrome	Common	Less common
Learning curve	Short (well-established)	Longer; Requires ultrasound training
Ultrasound compatibility	Technically feasible but limited acoustic window	Excellent visualization; ideal for real time guidance
Radiation exposure (Fluoro-only)	Moderate	Potentially higher if fluoroscopy only; Markedly reduced with ultrasound
Lead orientation	Angulated, potential stress	Straighter—Reduced mechanical tension and improved longevity

**Table 3:** Key Comparative Studies of Axillary vs. Subclavian (and Cephalic) Venous Access for CIED Implantation. Abbreviations: AP: axillary puncture; AVP: axillary vein puncture; CIED: cardiac implantable electronic device; CRT: cardiac resynchronization therapy; CV/CVC: cephalic vein (cutdown); ICD: implantable cardioverter-defibrillator; IPUS-AVP: intra-pocket ultrasound-guided axillary vein puncture; PLF: pacemaker lead failure; PM: pacemaker; RCT: randomized controlled trial; SP: subclavian puncture; US: ultrasound; USAA: ultrasound-guided axillary access; USAX: ultrasound-guided axillary (vein access).

Study	Design and Number	Access Compared	Main Findings	Key Methodological Notes
Kirkfeldt et.al., 2012 [5]	Population based cohort; N = 28,860	Subclavian vs. cephalic cutdown	Subclavian puncture → 7.8—fold higher pneumothorax risk	Registry data; no data on US use; Operator/center variability
Sharma et. al., 2012 [10]	Prospective, single-center, nonrandomized study; N = 478	Fluoroscopy-guided axillary vs. landmark subclavian access	Comparable success; Higher first-pass success and no pneumothorax in axillary group	Nonrandomized; Single center; Short follow up
Liu et.al., 2016 [9]	Randomized controlled trial; N = 247	Optimized axillary vs. subclavian	Higher overall success and shorter access time with optimized axillary; fewer access complications	Single-center RCT; Short follow-up;
Chan et. al., 2017 [6]	Retrospective cohort; N = 409 (681 leads)	Axillary (contrast-guided) vs. Subclavian vs. Cephalic	Axillary access independently associated with lower lead failure vs. subclavian; AP and SP had similar success rates; CV markedly lower.	Retrospective, single-center; few PLF events; operator-selected access → selection bias; mechanisms of PLF not lab-confirmed; procedural time not consistently recorded.
Liccardo et.al., 2018 [21]	Randomized study; N = 174	US-guided axillary vs. Subclavian	Similar first-attempt success; Axillary succeeded when subclavian failed; Similar lead complications at 18 months	Single-center; Short follow-up; Limited power for rare events
Migliore et.al., 2020 [22]	Prospective cohort; N = 95	US-guided axillary vs. Fluoro-guided axillary	Similar success (~92%); US access dramatically reduced radiation; no complications in either group	Single-center; small sample; nonrandomized
Tagliari et.al., 2020 [43]	Prospective multicenter RCT; N = 88	US-guided axillary vs. cephalic vein cutdown	Axillary access had higher success (97.7% vs. 54.5%), fewer access-site changes, faster venous entry and shorter procedural time; complication rates similar.	Operators had no prior axillary experience; Small sample; Early-only outcomes.
Courtney et.al., 2022 [28]	Prospective RCT; N = 100	US-guided axillary vs. Conventional (mixed)	USAA had high success (94%); early longer access times improved with experience; fewer venograms; similar 30-day complications.	Single-center; mixed comparator; Pronounced learning curve.
Maffè et al., 2023 [7]	Prospective cohort; N = 130 (CIED implantation)	US-guided Axillary vs. Conventional Subclavian/Cephalic	US-Ax showed high success, low complication rates, and favorable safety profile	Single-center; observational; no randomization; limited long-term follow-up.
Charles et.al., 2023 [30]	RCT; N = 200	IPUS-AVP vs. Cephalic cutdown	IPUS-AVP had higher success (99% vs. 87%) and shorter access, procedure, and fluoroscopy times; similar complications.	Single-center; Few operators; Short follow-up; Excluded upgrades/CRT; COVID-related recruitment limits.
Vitali et. al., 2024 [25]	Prospective RCT; N = 270	US-guided Axillary vs. Fluoro-guided Axillary	US-guided access reduced composite complications; markedly fewer arterial punctures; lower radiation exposure; similar first-attempt success	Single-center; high crossover rate; Mostly pacemaker patients (few ICD/CRT); Short 30-day follow-up.
Leventopoulos et.al., 2024 [27]	RCT; N = 114	US-guided axillary vs. Cephalic	Higher success with USAX; Shorter procedure time; Less pain; Similar complications	Single-center, two operators; operators less experienced in USAX; CRT/upgrades excluded; only short in-hospital follow-up; secondary endpoints exploratory.

## Data Availability

The original contributions presented in this study are included in the article. Further inquiries can be directed to the corresponding author.

## Abbreviations

The following abbreviations are used in this manuscript:

ACC	American College of Cardiology
AHA	American Heart Association
AI	Artificial intelligence
AP	Axillary Puncture
AV	Arteriovenous
AVB	Atrioventricular block
AVP	Axillary Vein Puncture
BMI	Body Mass Index
CIED	Cardiac implantable electronic devices
CKD	Chronic kidney disease
CT	Computed tomography
CV	Cephalic vein
CVC	Central venous catheter
ECMO	Extracorporeal Membrane Oxygenation
ESC	European Society of Cardiology
ESRD	End Stage Renal Disease
HRS	Heart Rhythm Society
ICD	Implantable Cardioverter-Defibrillator
IPUS-AVP	Intra-Pocket Ultrasound-Guided Axillary Vein Puncture
LPMs	Leadless Pacemakers
PLF	Pacemaker Lead Failure
PM	Pacemaker
RCT	Randomized clinical trial
S-ICD	Subcutaneous implantable cardioverter-defibrillator
SP	Subclavian Puncture
SVC	Superior vena cava
US	Ultrasound
USAA	Ultrasound-Guided Axillary Access
TAVR	Transcatheter aortic valve replacement
TLE	Transvenous lead extraction
